# Supplementary material for: Are carbon emissions trading and green financial instruments synergistic? -Comprehensive quantitative research based on content analysis
Source: PLoS One. 2024 Mar 7;19(3):e0298601. doi: 10.1371/journal.pone.0298601 (PMC10919601; doi:10.1371/journal.pone.0298601)
Supplement: S1 Data — (ZIP) [file pone.0298601.s001.zip › Data/地级市Z_F工作报告.zip!/║■▒▒/╛ú├┼╩╨/└┤╫╘╣1⁄2╓┌║┼í╛┬φ┐╦╩2╛▌═°í┐_2003.docx]

现在，我代表荆门市第五届人民政府向大会作政府工作报告，请予审议。并请市政协各位委员提出意见。

　　一、过去五年工作的回顾

　　本届政府任期，是荆门发展历程中不平常的五年，是全市人民在错综复杂的形势下艰苦奋进、在前所未有的困难中加快发展的五年。五年来，本届政府在省委、省政府和市委的领导下，在市人大、市政协的监督、支持下，团结和依靠全市人民，解放思想，抢抓机遇，加快发展，经受住了亚洲金融危机的冲击和各种自然灾害的频繁挑战，经受住了市场约束加剧、各方面矛盾增多的严峻考验，较好地 完成了市五届人大提出的各项工作任务。

　　（一）经济结构战略性调整取得成效，国民经济持续稳定增长。五年间，全市国内生产总值年均增长7.9%，顺利地完成了“九五”计划，并实现了“十五”计划的良好开局。到2002年，国内生产总值达到293.5亿元。

　　经济结构战略性调整取得成效，三次产业结构由1997年32：36：32调整为2002年21：44：35 ，二、三产业成为国民经济主体。

　　农业基础地位继续加强。围绕农民增收目标，推进农业结构调整和产业化经营，实施“六优一种”工程，不断改善农业生产条件，农村经济稳定发展。除粮棉因主动调减面积产量有所下降外，优质水稻、油料、水产、肉类、水果、蔬菜、食用菌等产品产量均有较大幅度增加。多种经营产值占农业总产值比重达70%以上。农产品优质率达60%以上。乡镇企业保持较快发展势头，增加值年均增长13.5%。

　　工业在改革和调整中平稳增长。工业增加值年均增长9.3%。

　　坚持扶优扶强，努力改善工业结构，推进技术创新，加强市场开拓，重点企业和主导产品支撑作用更加突出，化工、食品、建材、机电等支柱产业进一步发展。集中力量，狠抓荆门高新区建设和企业技 术进步，高新技术产业发展势头良好。实施“精品名牌”战略，“金龙泉”啤酒荣获全省唯一“中国名牌”。

　　第三产业发展较快。第三产业增加值年均增长9.7%。注重发展现代服务业，商贸业态不断创新，信息、通信、中介、房地产、金融、保险等行业发展迅速；全市旅游资源得到合理开发和有效整合，旅游业成为第三产业发展的亮点。

　　重点项目建设成效显著。五年累计完成固定资产投资290.5亿元，年均增长11.3%。农业、能源、交通、电力等基础设施建设加快。荆门石化聚丙烯、葛洲坝水泥厂四、五号窑扩建、金宇水泥厂迁建、百科药业甲醇、洋丰硫基氮磷钾、京山轻机瓦楞纸板生产线等重点项目开发与建设相继完成。汉江干堤加固、邓小两湖分洪区建设以及一批大中型水库整险加固等水利工程顺利实施，封山育林、退耕还林、水土保持等工程建设全面启动，全市森林覆盖率达到33.2%。市内交通状况大为改善，荆潜公路、皂当公路、汉宜公路、寺沙公路、安京公路、207国道复线均建成二级以上高等级公路，长荆铁路建设如期完成，襄荆高速公路建设，江汉航线建设，城乡电网改造，枣山220千伏变电站、500千伏三峡输变电工程，11万门程控电话安装、数字移动通信、邮件处理中心等重点项目建设进展顺利，为经济发展提供了有力支撑。

　　财政金融形势稳定。2002年，财政收入可达18.96亿元，年均增长5%；年末各类金融机构存款余额172亿元，贷款余额130亿元，分别比1997年增加74亿元和42亿元。妥善化解了城市信用社、农村合作基金会等金融风险。

　　（二）体制改革取得突破性进展，全方位开放格局逐步形成。坚持以国有企业改革为中心环节，推进资产重组和扭亏增盈，顺利完成了国有大中型企业改革三年脱困目标。全市共组建40家股份有限公司和46家企业集团，其中17家资产总额超亿元，4家进入全国重点联系的千家企业行列，新增2家上市公司和上市公司隶属企业。

　　全面推进市直中小企业产权制度改革，改制面达94%。国有资产监管、营运体系逐步形成。社会保障制度改革迈出较大步伐，我市被省政府确定为完善城镇社会保障体系唯一试点城市，养老、失业保险覆盖面继续扩大，城镇职工医疗保险制度改革成效明显。国家机关推行公务员制度和政府机构改革顺利完成。

　　适应经济发展和对外开放的需要，市内行政区划进行了适当调整，沙洋撤区设县，新设立了掇刀区和屈家岭管理区，顺利实施了乡镇管理体制改革。所有制结构得到优化，非公有制经济快速发展，全市个体私营经济产值（营业额）年均增长17.6%。

　　农村改革进一步深化，第二轮土地承包圆满完成，农村税费改革试点进展良好。部门预算、政府采购、会计委派等财政改革稳步推进，计划、投资、金融、住房、物价、粮棉流通和外贸体制改革都取得新进展。对外开放水平进一步提高，五年间，全市兴办外商投资企业49家，引进市外境内资金40亿元，实际利用外资累计1.7亿美元，累计完成出口3.1亿美元。对外经济技术合作取得突破性进展，百科药业在 巴基斯坦投资办厂。

　　（三）城市建设、管理水平不断提高，城镇面貌发生明显变化。用市场运作手段加快城市基础设施建设。五年间，中心城区累计投资11.5亿元，其中多渠道筹资近8亿元，完善了交通、通信、水、电、气等公共服务设施。老城区主要街道改造顺利完成，南城区道路建设、凤凰旅游区开发建设步伐加快；天然气主管网工程、广电通讯网络工程相继完工，天鹅广场、月亮湖广场、东宝广场先后建成；园丁小区一、二期，金宁居住区、石化小区等住宅小区投入使用，亿达世纪花园、葡萄园小区正在建设之中；经过综合整治的竹皮河已成为中心城区的一道景观。中心城区南扩进程加快，城区面积达到40平方公里，城市人口达33万。开展城市生态环境综合治理，绿化、美化、亮化工程成效显著，城市管理进一步强化，我市先后被评为“省级园林城市”、“全国城市环境综合整治优秀城市”和“全国造林绿化十佳城市”。以县城为龙头， 加快城镇化进程，小城镇建设规模和水平逐步提高。中心城区、钟祥城区、京山城区和胡集镇、后港镇、漳河镇分别夺得全省城镇规划建设管理“楚天杯”。

　　（四）精神文明建设迈出新步伐，科技、教育和各项社会事业全面发展。我市被授予“全国科技进步先进市”称号。经省批准，设立了“荆门高新技术开发区”。五年间，全市共完成各类科技计划538项，登记科技成果217项，12家企业被认定为国家和省、市高新技术企业，与武汉大学、武汉理工大学开展了市校合作。

　　科技对经济增长的贡献率达46%以上。教育改革和布局结构调整迈出较大步伐，素质教育全面推进，基础教育以县(市、区)为主的管理体制初步形成，职业教育和成人教育服务经济的功能进一步增强，高等教育成绩突出。社会科学、文化艺术、新闻出版、卫生体育、广播电视事业进一步繁荣。

　　《闹龙舟》获得中宣部“五个一”工程作品奖。

　　成功举办了“中国第五届民间艺术节暨湖北省第六届（金龙泉）啤酒节”、“中国国际舞狮邀请赛暨荆门市第五届运动会”。

　　文物保护工作进一步加强，明显陵被列为世界文化遗产。

　　加快卫生改革与发展，城乡居民健康状况得到改善。竞技体育成绩突出，群众性体育运动日益活跃。开展了第五次全国人口普查工作。计划生育工作连续四年在全省获一等奖。深入开展文明城市、文明行业、文明单位、文明村镇和文明社区创建活动。坚持不懈地加强和改进思想政治建设，“三讲”集中教育、“三个代表”学习教育活动和公民道德建设卓有成效，人们的思想观念和精神风貌发生了积极变化。驻荆部队、武警官兵在维护社会治安、支援地方建设、抢险救灾等方面发挥了重要作用。“双拥”工作、国防动员、民兵预备役、人防工作得到加强。国土资源管理、环境保护、劳动和保障、人事、审计、物价、统计、质量技术监督、工商管理、烟草专卖、药品监督管理、民政、民族宗教、对台事务、外事、侨务、气象、地方志、信访、保密、档案、老龄、 残疾人等工作取得新进步。

　　（五）为群众办实事成效明显，人民生活水平进一步提高。五年来，集中力量办成了一些符合人民群众意愿的事情，使人民群众得到了实惠。中心城区污水、粉尘、烟 尘污染得到有效治理，环境质量明显改善；多处市民休闲娱乐场所陆续建成开放；东宝山隧 道即将竣工；科技馆、图书馆、妇女儿童活动中心、青少年活动中心相继投入使用；广播电视“村村通”工程如期完成；连续三年多渠道筹资2274万元，基本解决了贫困地区5.94万人饮水困难；重视社会弱势群体的就业和生活保障，“两个确保”得到落实，全市有7．38万下岗失业人员先后实现再就业，有9.6万城镇居民纳入“低保”范围；五年投入扶贫帮困资金6500万元，解决了13万人的温饱问题；农村中小学教师工资兑现问题等得到了较好解决。

　　城乡居民收入逐年增加。2000年，全市总体达到小康水平。2002年全市城镇居民人均可支配 收入达到6480元，农民人均纯收入达到2999元，分别比1997年增长24.1%和6.7%。2002 年全市 社会消费品零售总额达到106.7亿元，年均增长8.1%。全市城镇人均居住面积由1997年 的10.9平方米增至2002年的15平方米。城乡居民人均储蓄存款4300元，比1997年增长75.5%。

　　（六）民主法制建设继续加强，政府职能转变加快。各级政府依法行政水平进一步提高。

　　市政府认真执行市人大及其常委会的决议，定期向市人大及其常委会报告工作并接受监督；十分注重市政协的政治协商、民主监督和参政议政；认真听取民主党派、工商联、党外人士和人民团体意见，促进了民主决策、科学决策。五年来，办复人大代表议案2件、建议552件，政协委员提案731件，办结率均为100%，满意和基本满意率达95%以上。始终把环境建设置于政府工作的突出位置，从政府部门入手，大力整治经济发展环境。

　　改革行政审批制度，建立了市行政服务中心、招投标交易中心和国库集中支付中心，行政效率和服务质量逐步提高。认真实行执法责任制和过错责任追究制，政府工作人员的法制观念 和依法办事能力增强。“三五”普法圆满结束。认真落实人民内部矛盾纠纷排查调处制度， 深入开展社会治安综合治理，与“法轮功”及“华南教会”等邪教组织的斗争取得重大胜利，社会治安持续稳定。积极推进政务公开、村务公开、厂务公开和校务公开，加大从源头预防和治理腐败的力度，广泛开展“纠风”及专项治理活动，推行经济责任审计，惩处了一 批腐败分子，廉政建设取得新的成绩。

　　各位代表！这些新的成就，无不浸透着全市人民的辛勤汗水，凝聚着社会各界的不懈努力 。在此，我代表市人民政府，向全市人民、驻荆部队、武警官兵、政法干警和在荆中央、省属各单位，向所有关心、支持和参与荆门建设的各界朋友，致以诚挚的敬意!对各位人大代表、政协委员给予政府工作的大力支持，表示衷心的感谢!

　　在肯定成绩的同时，我们也清醒地看到，在经济社会发展中仍然存在一些不可忽视的突出问题：影响经济发展的体制性、结构性矛盾依然突出，所有制结构不合理，经济运行的质量和效益不高，后劲乏力；农业产业化、农民组织化和农村城镇化水平不高，农民增收困难；工业产业结构、产品结构调整缓慢，经济外向度偏低；失业、待业人员增多，就业压力加大，社会保障体系还很脆弱，不稳定因素依然存在；政府管理体制和运作方式尚未得到根本转变，应对入世措施不多；投资环境有待进一步改善，一些政府部门和工作人员执政为民的意识有待强化，官僚主义、形式主义和消极腐败现象时有发生，等等。这些问题，已经引起我们的高度重视，将在今后工作中努力加以解决。

　　二、今后五年工作的奋斗目标和基本思路

　　2003年到2007年，是荆门全面推进经济结构战略性调整、完善社会主义市场经济体制和提高对外开放水平的关键时期，也是荆门全面建设小康社会、加快推进现代化建设的关键时期。 在这一承上启下、继往开来的重要时期，我们既面临着许多前所未有的机遇，又面对着十分严峻的挑战。为适应新形势，完成新任务，我们必须切实增强发展意识和忧患意识，解放思想，与时俱进，抓住机遇，加快发展，不断开创荆门改革开放和现代化建设的新局面。

　　今后五年，总的指导思想是：高举邓小平理论伟大旗帜，全面贯彻“三个代表”重 要思想，认真落实十六大精神和市第五次党代会精神，围绕全面建设小康社会目标，大力实 施“兴工富市”战略，推进结构创新、体制创新、科教创新和环境创新，提高工业化、信息 化水平，加快产业化、市场化、城市化进程，不断改进政府工作方式方法，努力使我市经济总量在省内位次前移，促进各项社会事业全面进步，为实现现代化建设第三步战略目标奠定 坚实基础。

　　今后五年的奋斗目标是：确保国民经济发展速度快于、经济增长质量高于、经济效益好于全省平均水平。到2007年，国内生产总值达到450亿元，年均增长8.5%；财政收入年均增长8 .5 %；全社会固定资产投资年均增长10%；社会消费品总额年均增长12%；外贸出口年均增长1 2%；实际利用外资年均增长10%；城镇居民可支配收入达到9000元，年均增长6%；农民人均纯收入达到3600元，年均增长3.5% 。

　　根据上述指导思想和奋斗目标，总结过去五年的经验，在今后的宏观决策和工作指导上，我们必须坚持解放思想，实事求是，与时俱进，把经济发展作为第一要务，从荆门实际出发，创新工作方法，做好“结合”文章，用发展的办法解决前进中的困难和问题；必须坚持以结构调整为主线，以改革开放和科技进步为动力，实施“兴工富市”战略，巩固农业基础地位，突破性发展第三产业，努力实现速度与结构、效益和后劲的统一；必须坚持发展社会主义市场经济，充分利用“两种资源”，开拓“两个市场”，全方位招商引资，提高经济外向度， 大力发展民营经济，增强县域经济实力；必须坚持转变政府职能，依法行政，从严治政，持之以恒地抓好环境建设，大力培育市场主 体，加强企业家队伍建设，在全社会形成鼓励人干事业，保护和支持人干成事业的良好氛围 ；必须坚持相信群众，依靠群众，走群众路线，注重解决关乎群众切身利益的热点、难点问 题，把提高人民生活水平作为根本目的；必须坚持“两手抓”，推进物质文明、政治文明、精神文明协调发展和可持续发展，把依法治市和以德治市结合起来，提高全民思想道德素质 ，开展文化创新，促进人的全面发展；必须坚持把政府工作置于党委领导和人大、政协监督之下，改革和完善决策机制，形成合力，促进各项事业更快更好地发展。

　　在具体工作中，要切实把握以下重点：

　　(一)大力调整优化经济结构，加快新型工业化进程。

　　推进工业化是全面建设小康社会的根本途径。

　　要坚持以信息化带动工业化，以工业化促进信息化，加快实施“兴工富市”战略，努力走出一条科技含量高、经济效益好、资源消耗低、环境污染少、人力资源优势得到充分发挥的新型工业化路子。在全市重点建设5个工业园区，即荆门高新技术产业区、京山工业园、沙洋纺织工业园、钟祥工业园、东宝建材工业园；形成七大产业群落，即以荆门石化为核心的 石油化工产业群，以荆襄磷化和洋丰集团为核心的磷化产业群，以葛洲坝水泥厂、经和陶瓷 为核心的建材产业群，以京山轻机、东光集团为核心的机械、电子产业群，以金龙泉、华联为核心的食品、饮料产业群，以百科药业为核心的医药工业产业群，以京山伟嘉、沙洋革基布、钟棉和众和纺织为核心的轻纺产业群。以高新技术产业区为龙头，以工业园区为载体，以产业群落为基础，以企业为主体，不断开拓工业经济新领域，提升产业素质和水平，实现荆门工业经济结构战略性调整。充分发挥中省企业的带动作用，大力支持和服务中省企业，继续实行“双向纳入”，共同发展。全面推进国 民经济和社会信息化，加强信息基础设施建设，积极发展电子政务、电子商务和企业信息化 、社会公共事务信息化，推动信息产业跨越式发展，构筑“数字荆门”。大力发展旅游业，全面开发大洪山风景区，充分发挥明显陵“世界文化遗产”的品牌效应，抓紧建设纪山楚文化旅游区，贯通旅游线路，使全市旅游景区、景点连线成片。

　　积极运用现代经营方式和服务技术改造传统服务业，加快发展金融、保险、咨询、中介、房地产、现代物流等现代服务业。通过大力发展二、三产业，改善经济结构，到2007年，全市三次产业结构调整为15：48：37。

　　（二）改进农村经济经营方式，加快农业产业化进程。农业产业化经营既是对农业生产经营方式的变革，又是对农村生产关系的进一步调整。要牢固树立“扶持产业化就是扶持农业，扶持龙头企业就是扶持农民”的观念，适应市场需求，发挥比较优势，依靠 科技进步，大力推进农业产业化。到2007年，全市农产品综合优质率达到85%，畜牧、水产 占农业经济比重超过50%。鼓励和引导社会资本、民间资本和外资参与农业产业化经营，结合荆门实际，围绕粮食、棉花、油料、畜禽、水产品、林果、蔬菜、黄姜、花卉苗木、食用菌等主要农产品 ，加快建立一批专业化生产、区域化布局的特色农业基地，重点发展和扶持一批与基 地、农户对接的龙头企业，促进农产品在产、加、销各个环节增值，实现农业增效，农民增收。

　　继续推进乡镇企业的结构调整、技术进步和体制创新，突出发展农产品加工业，延长产业链。坚持农业产业化与农村工业化有机结合、协调发展，形成贸工农一体化、产加销一条龙的农村经济经营方式和产业组织形式，推动农业产业化由低级形态向高级形态迈进，促进传统农业向现代农业转变，实现农业大市向经济强市跨越。

　　(三)继续推进改革开放，加快市场化进程。

　　今后五年，是深化改革、扩大开放，全面建立和完善社会主义市场经济体制的重要时期。要按照现代企业制度的要求，推进企业产权制度创新，完善法人治理结构，培育市场主体。推进国有资产管理体制改革，健全国有资产管理、监督和营运体系。

　　积极培育中介组织，大力发展产权、土地、劳动力和技术等生产要素市场，整顿市场经济秩序，活跃市场要素，规范市场行为，健全市场体系，全力打造“诚信荆门”。深化分配制度改革，合理调节收入 分配差距，提高知识、技术、资本参与收益分配的比重。建立独立于企事业单位之外，资金 来源多元化、保障制度规范化、管理服务社会化的社会保障体系。深化事业单位改革，继续 推进财税、金融、投融资、住房等各项改革。

　　坚持“引进来”、“走出去”和“联起来”，推进生产、贸易和资本参与国际经济大循环。 坚持利用外资与调整优化结构、促进产业升级相结合，扩大利用外资领域，创新招商引资方 式，提高利用外资的质量和水平。加强与世界知名企业、跨国公司的合作，提升我市企业整 体素质和参与国际竞争的能力。坚持以质取胜、科技兴贸和市场多元化战略，扩大出口规模 ，优化出口结构，提高出口效益。加强区域经济协作与交流，积极参与西部大开发，发展开 放型经济。

　　（四）促进非公有制经济快速发展，加快民营化进程。

　　非公有制经济是最具活力、最现实、见效最快的新的经济增长点。要继续大力调整所有制结构，积极鼓励和支持个体、私营经济加快发展，着力提高非公有制经济的比重和水平。进一 放宽民间资本的市场准入领域，实现公平竞争。取消对非国有投资限制性政策，大力培育多元化的社会投资主体。

　　鼓励和支持各种所有制经济相互参股和联合、兼并，形成国有资本进退联动和各种所有制经济相互融合、共同发展的机制。加快改革垄断行业，大胆引入民营机制，鼓励社会资本规范、有序进入。坚持“县域经济以民营经济为主”的方向，鼓励和帮助民营企业开展制度创新、技术创新和管理创新，重点培育发展一批销售收入过亿元的民营企业。

　　（五）建设现代生态园林城市，加快城市化进程。要以建设“生态园林城市”为目标，不断提高城市化水平。进一步完善市域城镇布局，加快中心城区、县（ 市）城区的建设和发展，积极培育小城镇，努力形成“一市多城、众星拱月”城市格局。到 2007年，荆门南城区的格局基本形成，中心城区建成区面积力争达到50平方公里，城市人口 达到43万人。

　　坚持走“经营城市”之路，提高城市建设的生态品位、文化品位和艺术品位，建设一批具有 现代化水平的标志性工程和城市精品，塑造优美的城市形象。

　　加强城市市政基础设施、公用服务设施和信息网络设施建设，加快路网、电网、燃气管网、供水管网、广播电视通讯网、信息网“六网”建设，提升城市综合服务功能。继续加强交通 基础设施建设，完成襄荆高速荆门段建设工程，抓紧荆宜高速荆门段开工建设，力争荆(荆 门)－东(东西湖)高速及早动工，争取经过京山的随（随州）－岳（岳阳)高速纳入国家建设 规划。境内主要干道建成二级以上公路，乡乡通油路，村村通公路。

　　坚持产业立镇、工业兴镇，努力建设一批基础产业好、服务功能全、建设品位高、带动能力强的小城镇。

　　牢固树立“环境效益”理念，实施城市环境综合治理工程，继续加大环境保护力度，采取有力措施，改善中心城区环境质量。

　　深化城市管理体制改革，推行综合执法，完善城镇管理体系和市场化运作机制，推进城市建设和管理现代化，形成以城带乡、以乡促城、城乡互动、共同繁荣的局面。

　　（六）大力发展先进文化，加快政治文明和精神文明进程。政治文明是社会主义文明认识的新境界，也是新世纪的新理念。要积极推进政治体制改革，扩大社会主义民主，健全 社会主义法制，实现社会主义民主政治的制度化、规范化和程序化。

　　坚持和不断完善社会主义民主制度，保证人民群众依法实行民主选举、民主决策、民主管理、民主监督。坚持依法行政，进一步规范政府行政行为，不断改进领导方式和执政方式，切实担当起改革、发展、稳定的重任。以人的全面发展为中心，进一步加强精神文明建设，积 极推进文化创新，努力形成先进的文化体制、现代的文化设施、发达的文化产业、繁荣的文化市场、丰富的文化生活，把荆门建设成“国家历史文化名城”。坚持“科教兴市”战略，加强技术创新体系建设，促进科技与经济紧密结合。加大教育投入，深化教育改革，开展教育创新，优化教育结构，合理配置教育资源，全面实施素质教育，加快普及高中阶段教 育。积极创造条件，争取本科院校申报成功。加快培育开放式终身教育体系，创建“学习型 ”社会。大力实施人才工程，加快人才资源开发，积极引进和培养高层次、高科技人才和复合型、创新型人才，构筑人才高地。

　　（七）切实转变政府职能，加快“服务型”政府建设进程。按照建立社会主义市场经济体制和WTO规则的要求，加快转变政府职能，切实将政府职能转到经济调节、市场监管、社会管理和公共服务上来，实现由管制型向服务型转变，由直接管理向间接管理转变，由行政管理为主向法制管理为主转变。深化行政管理体制改革，改进服务方式，减少行 政审批事项，实施“电子政务”，提高行政效率和服务水平，探索政府实行决策、执行、监督相分离的公共 管理模式，形成行为规范、运转协调、公正透明、廉洁高效的行政管理体制。

　　加强公务员队伍建设，优化结构，提高素质，增强依法行政和按国际规则办事的能力。坚持用硬措施改善软环境，把环境创新贯穿于经济社会发展的始终，进一步营造规范严明的法制环境、诚实守信的信用环境、便捷周到的服务环境、优美和谐的生态环境、安全稳定的社会环境，使荆门成为投资的热土。

　　（八）不断改善人民生活，加快全面建设小康社会进程。要不断增加城乡居 民收入，优化消费结构，拓宽消费领域，提高消费质量，让人民普遍享受基础教育、技能教 育、各种社会服务和现代化的城市基础设施服务，做到少有所教、老有所养、病有所医、安 居乐业。制定优惠政策，支持农民改造土坯房，到2007年城镇居民人均居住面积达到20平方 米，鼓励计算机进入家庭，提高电话普及率。

　　加强城乡公共设施建设，改善群众生活环境和出行条件。加大政府转移支付力度，提高低收 入家庭生活水平。

　　坚持就业优先原则，积极发展劳动密集型行业，广开就业门路，加大劳动力市场建设力度，完善再就业培训和职业技能训练体系，努力实现充分就业，每年新增城镇就业岗位1.5万个以上，城镇登记失业率在全省平均水平以下。建立适应新形势要求的卫生服务体系和医疗保健体系，切实改善农村医疗卫生状况，提高城乡居民医疗保健水平。完善多元化、社会化的投入机制，形成多渠道、多层次的社会 事业投入格局，发展城乡社会救济和社会福利事业。推进社区建设，逐步完善社区服务体系 。要带着深厚的感情关爱弱势群体，政府将采取积极措施，帮助他们解决就业、生活及子女就学等问题。继续开展扶贫帮困，巩固脱贫成果，消除绝对贫困。

　　三、2003年的主要工作2003年是全面贯彻党的十六大精神的第一年，也是新一届政府任期的第一年。做好开局之年的各项工作至关重要。我们要牢牢把握发展主题，在应对挑战中抢抓机遇，在扬长避短中增创优势，在超越自我中寻求突破，努力开创各项 工作的新局面。2003年全市国民经济和社会发展的主要预期目标是：国内生产总值增长9% 以上，全社会固定资产投资增长15%，财政收入按可比口径计算增长8.5%，利用外资增长20% ，外贸出口增长20%，社会消费品零售总额增长11%，城镇居民人均可支配收入增长5%，农民人均纯收入增加80元，城镇登记失业率控制在4.5%以内，全年新增就业岗位1.6万个，人口自然增长率控制在3‰以内。为实现上述目标，要扎扎实实做好以下十个方面的工作：

　　（一）提高工业运行质量和效益，增强工业经济整体竞争力

　　要坚定不移地大力实施“兴工富市”战略，坚持经济工作重点抓工业，工业重点抓投入，投入重点抓效益，以工业经济的快速、健康发展带活经济全局。

　　推进工业结构调整。开展“亿元工程”建设，以中省企业和地方骨干企业为核心，整合小而散和低水平重复的生产能力，扶持培育一批销售收入过亿元的企业。在水泥、化肥、食品加工、新型建材等行业建立和培育一批主业突出、科技含量高、竞争能力强的大集团、大公司。加大运用高新技术改造传统产业的力度，大力引导、支持企业推广机电一体化、先进制造等关键技术在传统产业中的应用，重点改造提高能源、化工、建材等产业，推进电子信息、新医药等新兴产业发展。突出抓好工业园区建设，加快工业布局调整和产业升级，充分发挥工业园区聚集效应。

　　狠抓工业项目建设。坚持实施重点项目“一把手”工程，重点抓好荆门石化成品油质 量升级工程、百科药业巴柳氮纳原料及颗粒剂和阿里普酶项目、荆襄磷化集团复合肥项目、钟祥减震器生产线、宝源木业高中密度纤维板生产线等项目建设。加大后劲项目开发力度，抓好项目包装。多渠道筹措工业发展资金，建立市、 县（市、区）两级工业发展专项资金。推进上市公司的资产重组和资本运营，培育高质量的上市后备 队伍。抓住国家继续实施积极财政政策的机遇，积极做好国债资金、高新技术产业化引导资金等国内外贷款的争取工作。

　　精心培植精品名牌。重点抓好金龙泉啤酒、三王牌包装机械、爱百颗药品等产品的升级和发展。加大名牌保护力度，树立 荆门名优产品的整体形象。积极开展管理创新。以人力资源管理为核心，吸 收国外企业的先进管理理念，创新管理思维，结合企业实际，规范企业各项管理制度，促进企业在科学管理中求生存、谋发展、增效益。

　　(二)全面发展农业和农村经济，切实增加农民收入

　　增加农民收入，繁荣农村经济，是农业和农村工作的中心任务。要在农业产业化、农村工业化、农村城镇化和劳动力转移等方面狠下功夫，寻求突破。培育特色农业。

　　以特求优，以特求效，引导农民继续调减粮食作物种植面积，稳定棉花、蔬菜、水果种植面 积，扩大“双低”油菜、花生种植面积，加快发展畜牧业、水产业，积极发展花卉苗木和 药材，建立和壮大具有地方特色和比较优势的种养基地和农产品出口基地。

　　继续实施“六优一种”工程，优化品种，提高品质。加快农业科技进步，建立农业标准化生产体系。

　　积极发展规模化生产，推行一乡一品，数乡一业，大力发展无公害食品、绿色食品和有机食品，争创国内外农业品牌。

　　推进农业产业化经营。从资金、技术、人才等方面，重点扶持国宝桥米、金林木业、华联蔬菜、鑫源食品、玉树色拉油等一批龙头企业，不断提高农产品加工、保鲜、贮运能力和 水平。完善“市场牵龙头、龙头带基地、基地连农户”的产业化发展模式，大力发展订单农 业。加快农产品批发市场向超市经营延伸。办好农村专业合作社、农村综合服务社等各类营 销中介组织和农民专业合作经济组织，

　　壮大农民经纪人队伍。发展非农产业。推进乡镇企业二次创业和产业升级；积极培育和完善农产品批发市场和特色专业市场，引导农民进入流通领域；大力发展“打工 经济”，跨地域、全方位地扩大劳务输出。

　　改善农业生产条件。充分利用“绿箱政策”，多渠道筹措建设资金，切实加大对农业的投入。加强农田水利基本建设，抓好节水灌溉和农业综合开发，积极发展避灾农业。继续抓好汉江堤防建设、病险水库整治和小流域综合治理。加大封山育林、退耕还林和天然林保护力度，加快宜林荒山荒地造林步伐，防止水土流失。坚持开发式扶贫方针，巩固扶贫攻坚成果。

　　（三）加快第三产业发展，促进消费需求增长

　　把加快发展和提高服务业作为调整经济结构、扩大社会就业和拉动内需的重要举措，努力提 高第三产业在国民经济中的比重。

　　大力发展旅游业。确立旅游业在三产业中优先发展的地位。以创建中国优秀 旅游城市为目标，加大旅游项目招商引资力度，加速景区景点开发建设，重点开发建设“三区”（明显陵-大洪山旅游区、漳河旅游度假区 、纪山楚文化旅游区）、“三山”（象山、圣境山和东宝山）和“一馆一楼”（博物馆、白云楼），形成“主打品牌 ”。

　　大力发展信息、咨询、社区服务、现代物流和商贸流通业。推动社会公共服务信息化建设，促进文化、教育、卫生等领域的信息资源开发和信息技术应用。

　　完善社区服务设施，发展社区服务业，积极开展家政、医疗等有偿服务。加强各类专业市场 和物流基地建设，发展特色商业街区，扩大农村连锁商业网点，发展超市连锁配送等新型商业业态。

　　大力发展房地产业。实施住房货币化分配，完善房地产二级市场政策，降低交易成本，扩大住房信贷，加快面向中低收入家庭的住房建 设，发挥房地产对扩大投资和促进消费的拉动作用。

　　（四）深化各项改革，增强经济发展动力

　　必须坚持以改革促发展，坚决冲破一切妨碍发展的思想观念，坚决改变一切束缚发展 的做法和规定，坚决革除一切影响发展的体制弊端，不断开拓促进先进生产力发展的新途径。

　　坚持深化国有企业改革。所有改制企业都要真正建立起“产权清晰、权责明确、政企分开、管理科学”的现代企业制度。按照“各司其责、协调运转、有效制衡”的要求，建立和完善法人治理结构。推行国有资产经营责任制考核和重大决策失误追究制，落实 企业经营者年薪制。

　　妥善处理好改制企业的遗留问题。加强国有资产管理、监督和运营，进一步提高国有资产的流动性。积极探索行政事业单位国有资产管理方式。

　　着力调整所有制结构。要大力发展多种所有制经济。放开准入领域，放宽准入条件，放手发展民营经济。在投资立项、用地用电、信贷支持、税费负担等方面，依法保障民营经济应有的“国民待遇”。鼓励民营企业采用资本注入、技术转让等方式，参与国有企业产权流动、资产重组和结构调整，实施规模扩张。积极支持私营企业申报进出口经营权。建立和完善民营企业融资信用担保体系，重点支持规模型、科技型、外向型民营企业。开展“十佳民营企业”评 选活动，激发民营企业家创业热情。鼓励机关事业单位干部职工领办、兴办民营企业。

　　加快建立和完善社会保障体系。抓好完善城镇社会保障体系的试点工作，继续落实“ 两个确保”，做好“三条保障线”的衔接工作。按照应保尽保的原则，将城市贫困居民全部纳入最低生活保障范围。调整养老保险、医疗保险缴费比例，调整财政性社会保障支出结构，提高社保覆盖面，抓好社会保险费征收以及社会保障资金的管理、运营、监督工作。积极稳妥地做好下岗职工协议期满出中心工作，逐步实现下岗职工基本生活保障与失业保险并轨。

　　千方百计援助弱势群体就业，把改善创业环境和增加就业岗位作为政府工作的重点，落实扩大城镇就业的优惠政策，实行多种形式就业。培育和发展劳务中介组织，加强就业培训和就业指导。进一步发展社会福利、社会救济、优抚安置和社会互助、法律援助等社会保障事业。

　　深化农村税费改革和各项配套改革。扎实推进农村税费改革，坚持“多予、少取 、放活”的方针，认真落实减人、减事、减支的各项措施，切实减轻农民负担。推进乡镇事 业单位改革、县乡财政体制改革，合理划分县乡事权、财权，确保农村义务教育支出和乡村基层政权正常运转。进一步深化粮食、 棉花、农资流通体制改革。探索土地流转机制，推进土地适度规模经营。采取有效方式化解村级债务。

　　（五）大力发展开放型经济，构筑对外开放新优势

　　加大招商引资力度。抓住国际产业和资本加速向内地转移的机遇，加大基础 设施、农业和服务业利用外资力度，鼓励外资参与国有经济调整和国有企业改组。坚持大员 上阵抓招商，舍得用优势企业、优良资产、优质品牌招商引资。进一步改进招商引资方式，实行政府招商与全民招商相结合，突出企业招商主体地位。进一步加强招商引资队伍建设，探索专业化招商新途 径。充分利用网络招商、中介招商等渠道，扩大招商引资范围。努力办好现有“三资”企业 ，以外引外。

　　继续落实招商引资目标责任制。高标准建设开发区，充分发挥各类开发区在招商引资中的带动作用。千方百计扩大出口。创新贸易方式，培育壮大机械、电子、绿色食品等传统出口产品 。继续扶持东光、百科亨迪、京山轻机、山塔铁合金、永祥、力新等重点出口企业。引导和支持出口企业降低成本，落实税收、信贷 优惠政策，增强出口产品竞争力。鼓励和支持企业开展质量管理体系、环境管理体系及各类产品认证活动。大力扶持出口导向型企业，培育壮大出口主体队伍，加快国有外贸企业改革 步伐。

　　加快实施“走出去”战略。加强与东南沿海地区的联合与合作，积极参与西部大开发。鼓励有条件的企业到境外投资办厂，积极开展 境外加工贸易、对外承包工程和劳务输出。

　　（六）加强城市建设和管理，促进城乡协调发展

　　突出抓好中心城区建设。按照“扩展南城区、完善西城区、提升东城区”的思路，引导基础设施建设向南城区转移。主要抓好“一区”（南城区东区）、“二场（厂）”（第二 垃圾处理场和南城区污水处理厂）、“三街”（襄沙北路、白云大道、荆钟路）、“三园” (东宝山公园、金龙泉植物园、楚文化园)、“六个居住区”（葡萄园居住区、广场周边居住区、金都花园居住区、果园居住区、亿达世纪花园、天鹅山庄）、“十条道路”(207国道城 区段和高新区段、阮家坪路、兴化大道、凤凰大道、果园一路和二路、象山大道南段、军马 场路、海慧路中段等道路的新建改造扩建)开发配套建设，以及东宝山隧道、三水厂管网配 套等 重点项目的后期建设。坚持创新城市建设投融资机制，吸引社会资本和外资参与城市建设。 完善土地收购储备制度，规范土地交易行为。加快供水、燃气等企业改制，推进市政、园林等城市公用事业改革。

　　进一步提高城市管理水平。大力开展城市环境综合整治，抓好市容卫生、交通秩序、 清临拆违、占道经营等专项整治，消灭脏乱死角，加大绿化力度，美化城市形象。坚持下移城市管理重心，逐步建立“统一领导、各司其职、规范管理、强化基层”的城市管理格局，形成依法管理、群众参与的城市长效管理机制。

　　加快交通基础设施建设。完成二级干线公路网建设，加快县乡快捷通道和乡 村公路建设，做好襄荆高速公路的协调工作，抓紧荆宜高速公路开工建设。

　　加大城镇建设力度。加强县（市）城基础设施建设，突出发展县城和中心镇，培植重点镇，建好口子镇。规范小城镇建设用地，深化城镇户籍制度改革，拓展投融资渠道，抓好小城镇综合改革试点，鼓励和支持农村人口向 小城镇集中。

　　(七)做好财税金融工作，促进经济健康发展

　　严格财政收支管理，推进财政各项改革。高度重视培财理财，按照“抓支柱、培骨干 、带群体”的财源建设思路，逐步形成结构合理、效益显著、后劲充足的地方财源新格局。 坚持依法治税，改进税务管理方式，强化税收征管，正确处理依法征税和优化经济环境的关 系，严厉打击骗税、偷税、逃税等各种涉税违法行为，确保应收尽收。完善国库集中收付制 度改革，建立科学的部门预算科目体系，加快公共财政建设步伐。强化预算约束，优化支出 结构，积极筹措和调度资金，保工资、保稳定、保改革、保重点，确保财政收支平衡。严格 控制非生产性建设，坚决压缩会议费、招待费等行政性支出，集中财力办好人民群众期盼的大事。加强财政监管，建立债务预警防范机制，提高财政资金 使用效率。充分发挥金融对地方经济发展的促进作用。稳步实施金融体制改革，推进金融创新。

　　进一步调整信贷结构，改善金融服务，信贷投入实行基础产业与工业并举、骨干企业与中小 企业并举、国有经济与民营经济并举。完善中小企业信用担保体系，创新适合各类市场主体 特点的贷款方式，加大金融支持经济发展的力度。以治理逃废金融债务为重点，以开展信用 等级评定为手段，营造良好的金融信用环境。加强金融监管，维护一方金融平安。

　　(八)实施“科教兴市”战略，提高国民经济整体素质和劳动者素质

　　大力推进科技创新，促进科技与经济紧密结合。以企业为主体，加强技术创新能力建设。继续推进产学研结合，抓好市校合作，支持企业与大专院校、科研院所结为经济共同体。推动有条件的科研和技术推广机构转向企业化经营。

　　继续加大政府科技投入，完善科技馆配套设施，加快建立以非政府投入为主的多元化科技投入体系，鼓励社会资本从事科技创业投资。

　　坚持优先发展教育，推进教育现代化。完善基础教育以县为主的管理体制，不断加大教育投入和贫困学生帮扶力度，巩固“九年制义务教育”成果，确保教师工资按时足额发放 。加大城区义务教育基础设施建设力度，加强高中学校建设，扩展优质教育服务面。加强职 业 教育和培训，优化、整合高校资源。建立健全多元办学体制，支持和规范民办教育。 加强教师队伍建设，培养一批具有较高 师德素养和业务水平的名教师，全面推进素质教育。加快教育信息技术建设，逐步实行教育 手段现代化。 推进学习型城市建设，构筑人才新高地。树立终身学习理念，建立多类学习型组织， 积极开展法律、外语、计算机、WTO规则和各种业务知识、专业技术的教育培训，提高全民 文化和科技素质。继续实施市“113”人才培育工程，重点培养和引进高新技术人才、高级经营管理人才、高级技能人才和参与国际竞争的人才。

　　高度重视企业家队伍建设，改善企业家创业环境，充分理解和尊重企业家的劳动和创造，培养和造就一支充满创业热情、具有创新精神和市场经济意识的企业家队伍。

　　完善知识、技术、管理等生产要素参与收益分配的激励政策，营造引才、荐才、聚才、用才 的良好环境。

　　(九)加强精神文明建设和民主法制建设，推进社会全面进步

　　以贯彻落实《公民道德建设实施纲要》为重点，大力加强精神文明建设。坚持用邓小平理论和“三个代表”重要思想教育广大干部群众，加强中国特色社会主义的共同理想教育 。以社会公德、职业道德、家庭美德为着力点，抓好市民思想道德建设，努力形成“爱国守法、明礼诚信、团结友善、勤俭自强、敬业奉献”的良好风尚。广泛开展形式多样、各具特色的精神文明创建工作，把创建文明社区、文明村镇、文明单位、文明家庭活动引向深入，从整体上提高城市文明程度和市民思想道德水准。

　　积极推进文化创新，加快发展各项社会事业。努力繁荣和发展文学艺术、社会科学、广播电视、新闻出版等事业，多出精品力作。深化文化体制改革，加快文化设施建设，大力 发展文化产业，活跃群众文化生活。认真做好中国第八届艺术节荆门分会场的申办工作。

　　加强文物保护工作。坚持不懈地“扫黄打非”，净化文化市场。开展全 民健身活动，提高竞 技体育水平，启动市体育馆工程建设。实施区域卫生规划，深化城镇医疗卫生改革，建立农 村医疗保障体制，提高预防保健和医疗服务水平。推进计划生育工作综合改革，稳定低生育 水平。加强环境保护和治理，促进人与自然和谐。做好妇女、儿童、残疾人、老年人工作， 维护和保障其合法权益。

　　加强民主法制建设，推进依法治市。自觉接受市人大及其常委会的监督，尊重人民代 表的民主权利。加强与人民政协的政治协商，接受民主监督，积极支持政协委员参政议政。 征求、集纳民主党派和社会各界人士意见，扩大政府决策及实施的社会基础。认真办理人大 代表议案、建议和政协提案。进一步扩大基层民主，完善村民自治和城市居民自治。积极实 施“四五”普法，提高公民法律素质，创造良好的法制环境，切实做到政府机关依法行政， 执法机关公正执法，企业依法经营，社会依法管理。

　　强化社会治安综合治理，维护政治稳定和社会安定。坚持“打防结合、预防为主”， 深入开展“打 黑除恶”等专项整治斗争，依法严厉打击严重刑事犯罪和经济犯罪。深化与 邪教组织的斗争，继续做好对“法轮功”痴迷者的教育转化工作。深入开展基层安全创建工 作，积极探索“打防控”长效工作机制。严格执行安全生产责任制和责任追究制，切实加强劳动保护，防止重特大安全事故发生。继续加强信访工作和“包保”责任制，妥善 处理人民内部矛盾特别是涉及群众切身利益的矛盾。认真抓好民族宗教工作。加强全民国防 教育，增强国防观念，做好“双拥”、民兵预备役和人防工作。

　　(十)建设服务型政府，提高政府施政效能强化服务理念，转变政府职能。

　　新一届政府要自觉推进服务型政府建设，树立服务为本、执政为民的理念，把“人民政府为人民”的承诺，切实变成每一名政府工作人员的实际行动。

　　进一步理顺政府、企业、市场的关系，实行政企、政事分开。把下级政府能办的事项，主动放下去；应由企业自主决定的投资决策、生产经营等事项，还权于企业；把社会可以承担的 事项，交给社会中介组织；属于竞争领域的资源配置，由市场机制去调节。继续抓好政府规 章制度的清理、修订和废止工作，深化行政审批制度改革，促成政府部门与中介机构彻底脱 钩，加快事业单位改革。要改变工作方式，努力掌握和综合运用经济、行政特别是法律手段管理经济和社会事务。规范行政行为，优化发展环境。

　　认真落实行政执法责任制和行政过错责任追究制，抓好执法队伍建设，提高依法行政水平， 切实解决多头多层执法、重收费轻服务等问题。坚持推行政务公开，构建电子政府，加强市 行政服务中心、国库集中收付中心和招投标交易中心建设。全面落实“收支两条线”管理规定，推行罚没事项集中审核、综合执行办法。强化市场监管，严厉打击制假售假、偷税骗税 等破坏市场经济秩序行为。倡导信用准则和诚信理念，加快社会信用体系建设，树立良好的 政府信用形象。加强政务督查，确保政令畅通。认真开展“市场主体评环境”活动。

　　加强政风建设，坚持勤政廉政。全体政府工作人员都要牢记全心全意为人民服务的宗旨，身体力行“三个代表”重要思想，按照作风建设“八个坚持、八个反对”的要求，保证把人民赋予的权力用来为人民谋利益。

　　大力提倡一线工作方法，在调查研究中完善思路，在深入群众中体察实情，在矛盾交汇处解 决问题。坚决克服奢侈浪费之风、文山会海之害、忙于应酬之俗、虚报浮夸之弊，以多办实 事、真抓落实、善用民智、深得民心的标准考察干部、评价政绩。认真落实廉政建设责任制 ，严格执行领导干部廉洁自律各项规定，切实加强群众监督、组织监督和舆论监督，抓好源头预防工作，加大案件查处力度，努力形成清正廉洁的为政之风。

　　发扬优良传统，坚持艰苦奋斗。艰苦奋斗是我党的传家宝。“艰难困苦，玉汝于成。”荆门要发达、要振兴，贵在艰苦奋斗。我们必须清醒的看到，荆门还不是很富 裕，条件还不是很好，还有大量的事情要做，相当一部分弱势群体还处于低生活水平。我们决不能贪图享乐，大手大脚。要牢记人民的重托和历史的责任，牢固树立为党和人民的事业 长期艰苦奋斗的思想，把艰苦奋斗贯穿于改革开放和现代化建设的全过程，与广大人民群众同甘共苦。

　　各位代表，党的十六大绘就了全面建设小康社会的宏伟蓝图，让我们在十六大精神指引下， 在市委的领导下，紧紧依靠全市人民，解放思想，振奋精神，再接再厉，开拓前进，共创荆门更加美好的未来！
